# IgE Antibodies against Cancer: Efficacy and Safety

**DOI:** 10.3390/antib9040055

**Published:** 2020-10-16

**Authors:** Jitesh Chauhan, Alex J. McCraw, Mano Nakamura, Gabriel Osborn, Heng Sheng Sow, Vivienne F. Cox, Chara Stavraka, Debra H. Josephs, James F. Spicer, Sophia N. Karagiannis, Heather J. Bax

**Affiliations:** 1 St. John’s Institute of Dermatology, School of Basic & Medical Biosciences, King’s College London, London SE1 9RT, UK; jitesh.chauhan@kcl.ac.uk (J.C.); alexa.mccraw@kcl.ac.uk (A.J.M.); mano.nakamura@kcl.ac.uk (M.N.); gabriel.osborn@kcl.ac.uk (G.O.); hengsheng.sow@kcl.ac.uk (H.S.S.); chara.stavraka@kcl.ac.uk (C.S.); debra.josephs@kcl.ac.uk (D.H.J.); sophia.karagiannis@kcl.ac.uk (S.N.K.); 2 School of Cancer & Pharmaceutical Sciences, King’s College London, Guy’s Hospital, London SE1 9RT, UK; james.spicer@kcl.ac.uk; 3 Epsilogen Ltd., London BioScience Innovation Centre, 2 Royal College Street, London NW1 0NH, UK; vivienne@epsilogen.com; 4 Guy’s Cancer Centre, Guy’s and St Thomas’ NHS Foundation Trust, London SE1 9RT, UK; 5 Breast Cancer Now Research Unit, School of Cancer & Pharmaceutical Sciences, King’s College London, Guy’s Hospital, London SE1 9RT, UK

**Keywords:** IgE, antibodies, cancer, immunotherapy, AllergoOncology, safety, type I hypersensitivity, anaphylaxis, in vivo models, basophil activation test (BAT)

## Abstract

Immunoglobulin E (IgE) antibodies are well known for their role in allergic diseases and for contributions to antiparasitic immune responses. Properties of this antibody class that mediate powerful effector functions may be redirected for the treatment of solid tumours. This has led to the rise of a new class of therapeutic antibodies to complement the armamentarium of approved tumour targeting antibodies, which to date are all IgG class. The perceived risk of type I hypersensitivity reactions following administration of IgE has necessitated particular consideration in the development of these therapeutic agents. Here, we bring together the properties of IgE antibodies pivotal to the hypothesis for superior antitumour activity compared to IgG, observations of in vitro and in vivo efficacy and mechanisms of action, and a focus on the safety considerations for this novel class of therapeutic agent. These include in vitro studies of potential hypersensitivity, selection of and observations from appropriate in vivo animal models and possible implications of the high degree of glycosylation of IgE. We also discuss the use of ex vivo predictive and monitoring clinical tools, as well as the risk mitigation steps employed in, and the preliminary outcomes from, the first-in-human clinical trial of a candidate anticancer IgE therapeutic.

## 1. Introduction

It has long been hypothesised that allergic disease and immunoglobulin E (IgE) may be protective against cancer. A number of epidemiological meta-analyses suggest that IgE sensitisation (allergies or atopy) may be correlated with lower risk of some malignancies, such as colorectal, glioma, gynaecological cancer, pancreatic cancer and childhood leukaemia [1,2,3,4,5]. Furthermore, patients with cancer may develop antitumour IgE: greater numbers of IgE-expressing cells have been observed in head and neck tumours compared to normal mucosa [6] and an IgE derived from pancreatic cancer patients showed antitumoural activity in vitro [7]. Furthermore, it has recently been reported that FcεRI expression [8] and greater numbers of eosinophils and basophils, key IgE receptor (FcεR)-expressing effector cells [9,10,11], may be associated with improved outcomes for cancer patients. In mice, where it should be noted the human IgE system is not fully recapitulated and expression of the high-affinity IgE receptor (FcεRI) is only present in mast cells and basophils, a carcinogen-induced IgE response was protective against carcinogenesis [12], but conversely an inflammation-driven polyclonal IgE response promoted tumour outgrowth [13]. These studies suggested that the contributions of IgE in immunological protection from tumour growth may depend on the nature of the IgE antibodies and the specific microenvironment. Overall, these data suggest that IgE antibodies could play a role in immunosurvelliance and, if designed to recognise tumour-associated antigens, may serve as useful therapeutic agents for cancer patients.

Currently, IgG1, the subclass with the highest propensity for activating gamma receptors (FcγRs), are utilised for antibodies designed to engender Fc-mediated effects, whereas IgG4 or Fc mutations (such as Leu234Ala/Leu235Ala (LALA) or Leu234Ala/Leu235Ala/Pro329Gly (LALA-PG) [14]) are employed when these functions are not sought. In contrast, it has been hypothesised that, due to potent activities in allergy and responses to parasites, IgE antibodies may be directed against cancers by recruiting and activating aspects of patient immunity and the tumour microenvironment not normally accessed by IgG class antibodies. The unique properties of IgE antibodies, including their high affinity for cognate receptors, prolonged tissue residency, engagement of tumour-resident immune effector cells and orchestration of potent effector functions, may make them well-suited for this therapeutic use.

High affinity for cognate receptors: The affinity of IgE for its high-affinity receptor, FcεRI, is 100- to 10,000-fold higher than IgG subclasses for their FcγRs. The affinity of IgE for its low-affinity receptor, cluster of differentiation 23 (CD23), is 100- to 1000-fold lower than to FcεRI, and the avidity of IgE-CD23 binding is equivalent to IgG-FcγRI interactions [15,16]. The sensitisation of effector cells by IgE for immediate response, which is the hallmark of allergic responses, may also be a beneficial characteristic leading to potent and persistent immune effector functions in tumours. IgE can also bind the soluble receptors galectin-3 and galectin-9. Galectin-3 is recognised for its contribution to tumour progression and metastasis [17,18], whereas galectin-9 may mediate antiproliferative functions in the cancer context [19,20]. However, the roles of these receptors have not been studied in relation to anticancer IgE therapeutic candidates.

Absence of inhibitory Fcε receptors: The inhibitory signalling of FcγRIIb can hamper the efficacy of IgG1 antibodies. However, there are no known inhibitory FcεRs, meaning that IgE-mediated effector functions are not likely to be suppressed in tumours as are IgGs.

Longer tissue residence: IgE has a significantly shorter serum half-life (1–2 days) as compared to IgG (2–3 weeks) [21,22]. This is attributed to the lack of binding to the neonatal Fc Receptor (FcRn) [23], which is responsible in protecting IgG from catabolism. In contrast, the half-life of IgE in the tissues is significantly longer than IgG (~2 weeks for IgE, compared to 2–3 days for IgG) [24]. The long retention of IgE on immune cells with the potential to induce prolonged immune surveillance therefore has the potential for improving the treatment of solid tumours.

IgE immune effector cells in tumours: Immune effector cells that express FcεRI and CD23 are known to infiltrate the tumour microenvironment (TME). In humans, basophils and mast cells express high levels of the tetrameric αβγ2 FcεRI, while monocytes, macrophages, eosinophils and dendritic cells express the trimeric αγ2 FcεRI at lower levels. Furthermore, subsets of B cells express CD23, while monocytes and macrophages can be stimulated under Th2 conditions to upregulate CD23 [25]. Although, these cells can be tolerant of tumour growth and exert protumoural functions, when engaged by tumour antigen-specific IgE antibodies via FcεRs, powerful effector functions directed towards tumour may be harnessed [26].

Potent Fcε-mediated immune effector functions: The powerful functions of IgE when bound to its receptors, CD23 and FcεRI, and cross-linked on the surface of immune effector cells are well characterised in allergic disease. Interaction of the target tumour cells with IgE bound immune cells has been reported to lead to similar effects against cancer [27]. IgE-FcεRI-mediated effector functions include mast cell and basophil degranulation and release of proinflammatory cytokines and chemokines which can activate immune responses and recruitment of inflammatory cells into the TME [27,28]. Additionally, monocytes, macrophages, mast cells and eosinophils can mediate antibody-dependent cell-mediated cytotoxicity (ADCC) through the release of toxic mediators, such as nitric oxide, proteases and cytokines like tumour necrosis factor (TNFα), whereas macrophages and monocytes can additionally trigger antibody-dependent cell-mediated phagocytosis (ADCP) [29,30] with monocyte/macrophage-mediated ADCC and ADCP via FcεRI and CD23, respectively [31,32] (see Section 2.1 and Section 2.3). It is also plausible that the release of tumour antigens can form immune complexes with IgE which can be taken up by FcεR-expressing antigen-presenting cells (APCs), such as dendritic cells (DCs), B cells and macrophages, and facilitate antigen presentation and prime antitumour adaptive T cell responses, such as in draining lymph nodes [33,34].

Taken together, these attributes make IgE a molecule with much potential to target cancer. Therefore, pipelines to rapidly and cost-effectively clone, express and purify IgE antibodies for pre-clinical study have been developed [32,35,36,37,38,39] and indeed, in the field of AllergoOncology [25,26,34,40,41], in vitro and in vivo data (described below) have demonstrated the promising therapeutic efficacy of tumour-targeting IgE antibodies.

## 2. Pre-Clinical Efficacy of Antitumour IgE Antibodies

### 2.1. In Vitro Studies

IgE antibodies targeting several tumour-associated antigens have been studied for their Fab- and Fc-mediated functions in vitro (Table 1). Mouse/human chimeric IgE (MOv18) specific for the ovarian cancer antigen, folate receptor alpha (FRα)-mediated ADCC and ADCP of ovarian cancer cells (IGROV1) by human peripheral blood mononuclear cells (PBMCs) [29], human monocytic U937 cells [30] and peripheral blood monocytes and eosinophils [42]. Similarly, the MOv18 mouse/rat surrogate IgE (engineered for in vivo studies, as described in more detail below), mediated tumour cell ADCC by rat primary monocytes and U937 cells, and ADCP by IL-4-primed U937 cells with upregulated CD23 expression [32]. Likewise, ADCC was mediated by anti-epidermal growth factor receptor 2 (Her2) (trastuzumab variable regions) [36,43], anti-epidermal growth factor receptor 1 (EGFR) [37,44], anti-CD20 [45] and SF-25 [46] engineered IgE antibodies, and those from colorectal cancer patients [7]. Furthermore, anti-Her2 (C6MH3-B1 variable regions) and anti-prostate-specific antigen (PSA) IgE antibodies enhanced antigen presentation by DCs to induce CD4+ and CD8+ T cell activation [47,48]. Additionally, anti-Her2 (trastuzumab variable regions) and anti-EGFR antibodies elicited Fab-mediated direct effects inhibiting cancer cell proliferation and Her2 signalling [36,43,44]. Finally, cross-linking of receptor-bound IgE antibodies by polyclonal anti-IgE antibody, multimeric antigen, or antigen-expressing cancer cells triggered degranulation of human FcεRI-expressing rat basophilic leukaemia (RBL SX-38) mast cells [36,39,43,44,47,48,49].

### 2.2. In Vivo Studies

The therapeutic efficacy of IgE has been shown in several in vivo animal models (Table 2). Nagy et al. demonstrated that a murine IgE, against the major envelope glycoprotein (gp36) of the mammary tumour virus (MMTV), prevented the development of subcutaneous (s.c) and intraperitoneal (i.p.) H2712 mammary carcinoma in syngeneic C3H/HeJ mice [50]. Similarly, Kershaw et al. reported that a murine IgE, 30.6, inhibited the growth of s.c. COLO 205 colon carcinoma in severe combined immune deficient (SCID) mice, when the IgG counterpart did not. In contrast, mouse/human chimeric 30.6 IgE failed to elicit antitumour immunity [51]. This is expected as human IgE does not bind to mouse FcεRI [52]. Moreover, mouse IgE binds not only to mouse FcεRI but also activating mouse FcγRIV which is expressed by monocytes, macrophages, neutrophils, and dendritic cells [53]. It is therefore possible that the antitumour efficacy of the mouse IgE may be mediated by mouse FcγRIV and/or FcεRI. Moreover, murine studies with IgE antibodies are also complicated by the fact that FcεRI expression is restricted on some immune cell types (mast cells, basophils) as compared to human counterpart [52]. A syngeneic mouse system is therefore not a suitable model for studying IgE immunotherapy.

These factors can be overcome by using hFcεRI transgenic (Tg) mice. In the hFcεRI transgenic mice, FcεRI can be found on mast cells, basophils, monocytes, Langerhans cells, B cells and eosinophils. The expression pattern of FcεRI is similar to the human counterpart and therefore more suitable for studying the in vivo effects of tumour targeting IgE. In an in vivo mouse mammary tumour model, anti-MUC1 or anti-Her2 (C6MH3-B1) IgE therapy reduced mucin-1^+^ (MUC1^+^) or Her2^+^ tumour outgrowth, respectively, and improved the survival of tumour-bearing hFcεRI transgenic mice [45,47]. Furthermore, in the prophylactic setting, vaccination with anti-PSA IgE (AR47.47) and PSA complexes prolonged the survival of hFcεRI transgenic mice challenged with PSA-expressing CT26 tumour cells [48]. Taken together, the studies in hFcεRI transgenic mice suggest that IgE has the capacity to induce tumour cell death and trigger antitumour immunity by engaging hFcεRI expressing cells. However, as hFcεRI transgenic animals are not transgenic for CD23, the overall efficacy of the IgE may be underestimated [54].

Human PBMC-engrafted human xenograft tumour models have been used to study both FcεRI- and CD23-mediated antitumour responses. Gould et al. constructed mouse/human chimeric MOv18 IgE and IgG1 antibodies specific for the ovarian cancer antigen, FRα [31]. SCID mice, which were s.c. challenged with human IGROV1 ovarian carcinoma cells, were engrafted with human PBMCs and treated with either MOv18 IgE or IgG1. While tumour growth in IgG1 treated animals was initially restricted, by day 35 post-challenge tumour size was comparable to the controls. In contrast, at day 35 tumour growth was restricted by up to 72% in mice treated with PBMCs and MOv18 IgE. [31]. Having developed a patient-derived xenograft (PDX) model from the ascitic fluid of a stage 3 serous cystadenocarcinoma patient, and demonstrating that FRα expression was retained in these tumours, further efficacy studies were performed. Nude mice i.p. challenged with PDX donor mice ascites, and treated with human PBMCs and MOv18 IgE, showed prolonged survival compared to controls [29,42]. In the same model, administration of MOv18 IgE with human monocytic cells (U937), some of which had been pre-stimulated with IL-4 prior to injection, also prolonged mouse survival compared with those given effector cells alone [30].

Human PBMC-engrafted xenograft models have been invaluable for IgE immunotherapeutic studies; however, they are not a suitable toxicology model due to the short-life span of the allogenic human PBMC and the potential for graft-versus-host disease (GVHD) in recipient immunodeficient mice. Recently, an immunocompetent WAG rat model was designed to better recapitulate the human IgE-Fc biology system. The design of this model was based on the expression patterns and distribution of rat FcεRI and CD23, thought to closely mimic the distribution of human counterparts [55]. Expression of the trimeric FcεRI on monocytes, macrophages, DCs, eosinophils and other immune cells is similar in humans and rats, but not in mice. Therefore the rat was considered a superior model for IgE efficacy and toxicological studies, in which surrogate rat antibodies engage with rat immune effector cells in immunocompetent animals to target syngeneic rat tumour cells [32]. In a FRα-expressing rat CC531 carcinoma lung metastasis model in WAG rats, rat MOv18 IgE significantly reduced the number of, and area occupied by, lung metastases compared to animals treated with rat MOv18 IgG2b [32] (safety studies using this model are described in Section 3.3.3).

In summary, these results demonstrate the potential anti-tumour effects of IgE antibody therapy against a range of tumour targets and suggest that IgE class antibodies have the capacity to activate FcεR-expressing effector cells to induce tumour cell killing in vivo.

### 2.3. Monocytes and Macrophages are Key Effector Cells in the Mechanism of Action of Anti-Cancer IgE

In addition to demonstrating potential for therapeutic superiority of IgE isotype antibodies compared to IgG, findings from multiple rodent models of cancer, as well in vitro and ex vivo functional studies, support a monocyte and macrophage-driven effector mechanism for antitumour IgE.

Accounting for up to 50% of the mass of tumours, tumour-associated macrophages (TAMs) are highly abundant [56]. This principally occurs through potent upregulation of chemoattractants in the tumour microenvironment (TME) for circulating monocytes, including colony stimulating factor 1 (CSF-1) and monocyte chemoattractant protein-1 (MCP-1) [57]. Moreover, and crucially from a functional perspective, TAMs strongly exhibit the inherent phenotypic plasticity of macrophages, which facilitates their ability to promote a broad range of tumour supportive processes [58]. Macrophages are traditionally considered to display two distinct functional phenotypes following in vitro stimulation, defined as interferon-γ (IFN-γ) and lipopolysaccharide (LPS)-induced M1 macrophages and interleukin-4 (IL-4)-induced M2 macrophages, which exhibit proinflammatory/immunostimulatory and immunosuppressive/pro-repair phenotypes, respectively [59]. In vivo, macrophage plasticity extends beyond this M1/M2 axis, especially in the TME, where TAMs are simultaneously exposed to a diverse range of enriched stimuli, including hypoxia, type 2 cytokines such as IL-4, and anti-inflammatory and proinflammatory cytokines such as IL-10 and IL-6, respectively [60]. This generates a heterogenous TAM population, which overall is considered to display a predominant polarisation towards M2-associated immunosuppressive and pro-repair phenotypes, leading many to define these as M2-like [59]. Adoption of these phenotypes diminishes M1-associated antitumour functions, such as immune stimulation and tumour cell destruction, and instead promotes protumour processes such as tissue remodelling, metastasis and crucially, potent suppression of antitumour immunity [58]. This is typified by the prevalent IL-10^high^IL-12^low^ TAM subset observed in ovarian cancer, which promotes immunosuppressive cells such as regulatory T cells (Tregs) and myeloid derived suppressor cells (MDSCs) [61].

In most solid tumour types, such as breast [62], pancreatic and bladder cancer, high TAM density is generally a negative prognostic factor. However, in ovarian cancer, TAMs only correlate with worse disease outcomes, when a high density of M2-like macrophages are present (CD163+) [63]. In fact, an elevated ratio of M1-like (HLA-DR+) to M2-like macrophages actually predicts improved progression-free survival (PFS) in ovarian cancer patients [63]. These findings underline that, despite frequently being key drivers of tumour progression, TAMs are cells of immense phenotypic plasticity, capable of providing a net antitumour contribution to the TME, if stimulated appropriately.

**Figure 1:**
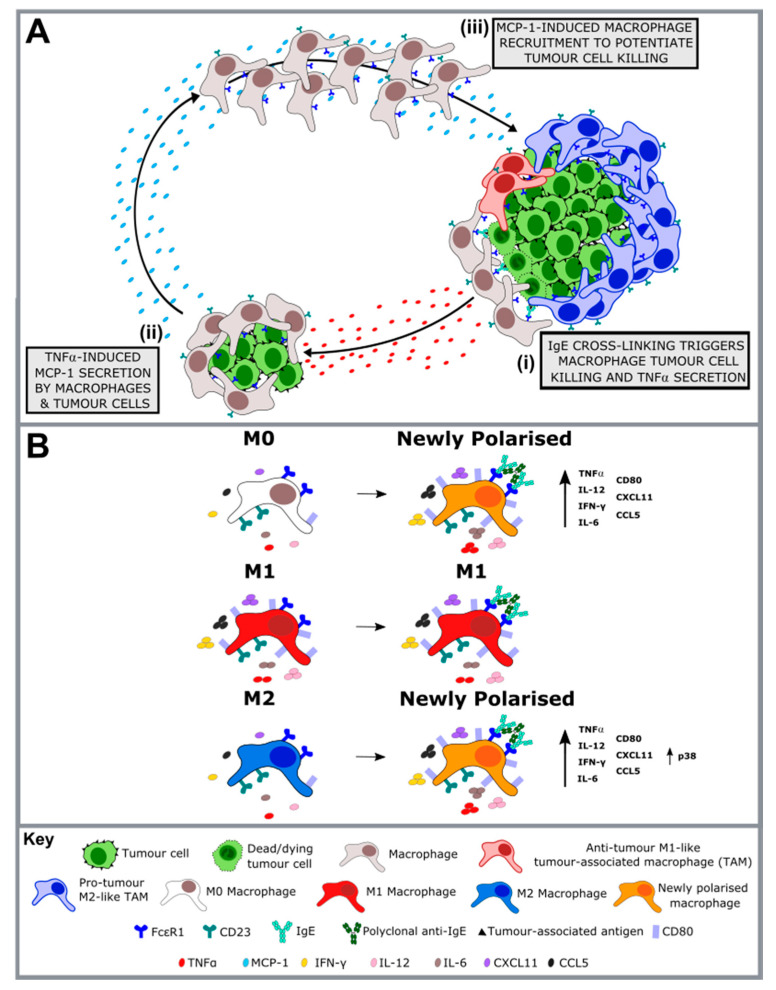
Cross-linking of IgE antibodies bound to monocyte and macrophage FcεRs by tumour-associated antigens (TAAs) is proposed to mediate antitumour activity via a two-armed effector mechanism. (**A**) First, IgE cross-linking activates monocytes and macrophages to induce both tumour cell killing, as well as a proinflammatory recruitment positive feedback loop, mediated by TNFa/MCP-1, which potentiates tumour cell death. Adapted from Josephs et al. [32]. (**B**) Second, IgE cross-linking (horizontal arrows) triggers a repolarisation of tumour associated macrophages (TAM) subsets that are otherwise protumourigenic. In vitro IgE cross-linking induces a proinflammatory and immunostimulatory phenotypic repolarisation of those monocyte-derived macrophage (MDM) subsets that are predominantly associated with protumour functions (M0 (unstimulated) and M2 (IL-4-stimulated)), while maintaining the phenotype of principally tumouricidal M1 (IFN-γ + LPS-stimulated) MDMs. Adapted from Pellizzari et al. [64].

Cross-linking of IgE antibodies bound to monocyte and macrophage FcεRs by tumour-associated antigens (TAAs), displays evidence of a two-armed antitumour mechanism for IgE: first, through monocyte and macrophage-mediated tumour cell killing, and second by repolarisation of tumour-supportive M2-like TAM subsets, through enhancement of proinflammatory and immunostimulatory activity (Figure 1).

In the Karagiannis et al. human xenograft model of a patient-derived ovarian carcinoma in immunocompromised mice, the survival benefit provided through treatment with MOv18 IgE was abrogated when engrafted human PBMCs were depleted of monocytes [42]. However, the survival benefit from MOv18 IgE was restored when depleted PBMCs were reconstituted with monocytes. Furthermore, in vitro studies of human monocytes, isolated from both healthy volunteers and cancer patients, as well as healthy volunteer human monocyte-derived macrophages (MDMs), have consistently exhibited IgE-mediated tumour cell cytotoxicity [32,46]. Both monocytes and macrophages display constitutive FcεRI expression, with the high affinity receptor mediating cytotoxic killing of cancer cells (ADCC) upon IgE engagement [31,32]. Moreover, monocytes and macrophages both display upregulation of CD23 following IL-4 stimulation, a cytokine which is frequently reported at high levels in the TME and which can drive M2-like TAM polarisation [57]. In vitro, the low-affinity receptor has been shown to mediate phagocytic killing (ADCP) of cancer cells upon IgE engagement, to augment overall IgE-mediate cancer cell killing [31,32]. Furthermore, in the Josephs et al. WAG rat lung metastasis model, animals treated with rat MOv18 IgE displayed greater infiltration of CD68+ macrophages into the tumour islets, relative to MOv18 IgG or PBS-treated animals [32]. These macrophages exhibited evidence of repolarisation, displaying upregulation of the T cell co-stimulation ligand, CD80, and the proinflammatory and immunostimulatory cytokine, TNFα. Moreover, IgE-treated rats exhibited higher levels of secreted TNFα and the monocyte/macrophage chemoattractant, monocyte chemoattractant protein 1 (MCP-1), in the bronchoalveolar lavage (BAL) fluid. In vitro cross-linking of MOv18 IgE-bound human monocytes by FRα-expressing IGROV1 ovarian carcinoma cells exhibited the same cytokine signature (TNFα, MCP-1) observed in vivo, with secretion and IGROV1 mRNA expression of MCP-1 inhibited by pretreatment with a TNFα receptor-blocking antibody [32].

Collectively, this has led to the proposal of the following mechanism for IgE-mediated tumour cell killing [32]. First, TAA-bound IgE cross-links FcεR on monocytes and macrophages, activating these cells and inducing tumour cell killing (Figure 1A(i)). Cross-linking activation also induces secretion of TNFα, to trigger a proinflammatory recruitment positive feedback loop. TNFα stimulates chemoattractant MCP-1 secretion both from tumour cells as well as monocytes and macrophages (Figure 1A(ii)), inducing intratumoural migration of additional monocytes and macrophages. Finally, following their IgE-cross-linking activation, these recruited monocytes and macrophages then mediate both further tumour cell killing and TNFα secretion, to potentiate the cycle (Figure 1A(iii)). High expression of this TNFα/MCP-1 signature has been found to be associated with improved 5-year overall survival in ovarian carcinoma patients, suggesting that enhancement of this monocyte and macrophage axis may have significant clinical relevance [32]. Interestingly, a similar immunological signature has been observed in IgE-dependent monocyte and macrophage parasite clearance.

Moreover, this proinflammatory macrophage recruitment feedback loop is considered to promote the second arm of the antitumour IgE mechanism: repolarisation of tumour supportive M2-like macrophage subsets [46]. In vitro analysis of human MDMs determined that cross-linking of a surface-bound anti-tumour IgE, SF-25, induced repolarisation of subsets predominantly associated with protumour immunosuppressive and pro-repair functions, namely, M0 (unstimulated) and M2 (IL-4-stimulated) MDMs. Moreover, the phenotype of principally tumouricidal M1 (IFN-γ + LPS-stimulated) MDMs was maintained [46]. In concordance with the upregulation of CD80 and TNFα displayed by MOv18 IgE-activated rat macrophages in tumour-bearing rats [32], both M0 and M2 human MDMs exhibited an upregulation of proinflammatory and immunostimulatory markers, following IgE cross-linking. These included CD80 and TNFα, IFN-γ, IL-12 and IL-6, as well as lymphocyte chemoattractants, C-X-C motif chemokine ligand 11 (CXCL11) and C-C motif chemokine ligand 5 (CCL5), to produce a newly polarised MDM phenotype which closely resembled M1 MDMs (Figure 1B). Furthermore, from an intracellular signalling perspective, M2 MDMs displayed a marked upregulation of Lyn kinase following IgE cross-linking, which is known to promote initiation of FcεRI signaling in mast cells and basophils [46]. Downstream of FcεRI/Lyn activation, M2 MDMs exhibited increased phosphorylation of various additional kinases, including mitogen-activated protein kinase (MAPK) p38 (Figure 1B). p38 activation has been well documented to promote proinflammatory macrophage polarisation. For example, constitutive macrophage p38 activation has been found to induce a similar phenotype to the observed IgE-activated phenotype, including enhanced secretion of the proinflammatory cytokines TNFα, IL-12, MCP-1, IL-6, IL-1β and granulocyte-macrophage colony-stimulating factor (GM-CSF) [65]. In contrast, inhibition of p38 blocks TNFα secretion in LPS-activated monocytic THP-1 cells [66].

In view of (i) numerous in vitro and in vivo observations that monocytes and macrophages drive anti-tumour IgE functions [31,32,42]; (ii) the consistent absence of upregulation of type 2 cytokines, such as IL-4, in these models [32,67]; and (iii) elevation of molecules associated with type 1 immune responses, including IFN-γ, IL-12, TNFα, IL-6, CXCL11 and CCL5 following IgE cross-linking on MDMs [46], this axis more closely resembles an IgE-mediated parasiticidal response, rather than an IgE-mediated allergic profile [68,69]. Taken together, these findings suggest that, in addition to inducing direct tumour cell killing, IgE may restore immunostimulatory activity of TAMs. This functional repolarisation may consequently promote a shift in the TME towards tumouricidal inflammation, through enhanced recruitment and activation of antitumour cells, such as Th1, CD8 and NK cells, and suppression of protumour cells, such as Tregs and MDSCs.

As described above, other FcεRI and CD23-expressing immune effector cells such as mast cells, basophils, eosinophils and dendritic cells can also be engaged by tumour-targeting IgE antibodies. Mast cells and dendritic cells have been shown to elicit potentially anti-tumoral functions in vitro, such as degranulation and antigen presentation, respectively (see Section 2.1 and Table 1). It is possible that these cells could also be activated by IgE cross-linking to release factors, such as IL-4, IL-5, IL-13, VEGF and hepatic growth factor, into the TME which could be protumoral by promoting angiogenesis, antagonising antitumoral activities of monocytes/macrophages, or interacting with Treg cells [70,71,72,73]. However, no elevation in the levels of such mediators, including IL-4, IL-5, IL-6 and IL-8, following treatment with antitumour IgE in in vitro and in vivo models of cancer has been measured to date [32,67] and the number of these cells in the TME is significantly lower compared to monocytes/macrophages.

## 3. Considering the Safety of Anti-Cancer IgE Therapeutics

### 3.1. IgE and Type I Hypersensitivity

Upon re-exposure of the immune system to the same allergen, a two-phase immune response takes place. The first phase is the early phase reaction, consisting of immediate type I hypersensitivity, followed by the late phase reaction that takes place within 4–6 h after the early phase reactions, and can last for weeks [74]. Type I hypersensitivity can occur locally, at the site of antigen challenge, or systemically, which could potentially result in systemic anaphylaxis, a severe and potentially life-threatening reaction caused by a sudden and acute degranulation of mast cells and basophils resulting in a multiple of systemic symptoms including erythema, hypotension and/or cardiac arrest [74]. Type I hypersensitivity is triggered by the cross-linking of monoclonal IgE antibodies that are bound on the FcεRI of mast cells or basophils via engagement with a multivalent antigen, allowing interaction of two or more receptors simultaneously which causes the activation of downstream intracellular signalling cascades. The only exception to this is cytokinergic IgE antibodies which are capable of activating immune effector cells in absence of cross-linking by multivalent antigen, but have a propensity for self-association and formation of antibody trimers which drive activity [75,76,77]. The aggregation of cross-linked FcεRI-IgE instigates mast cell or basophil degranulation, triggering a release of multiple soluble mediators such as histamine, serotonin, heparin, proteases and lipid mediators, leading to immediate allergic symptoms [74,78].

Although there have been no reports of anaphylaxis in individuals with naturally occurring tumour specific IgE antibodies [6,7], a significant challenge in the development of therapeutic anticancer IgE antibodies has been the consideration of this perceived risk of hypersensitivity. Therefore, a number of in vitro and ex vivo safety and toxicity assessments have been conducted.

### 3.2. In Vitro Studies of Potential Hypersensitivity Reactions to Therapeutic IgE

**Figure 2:**
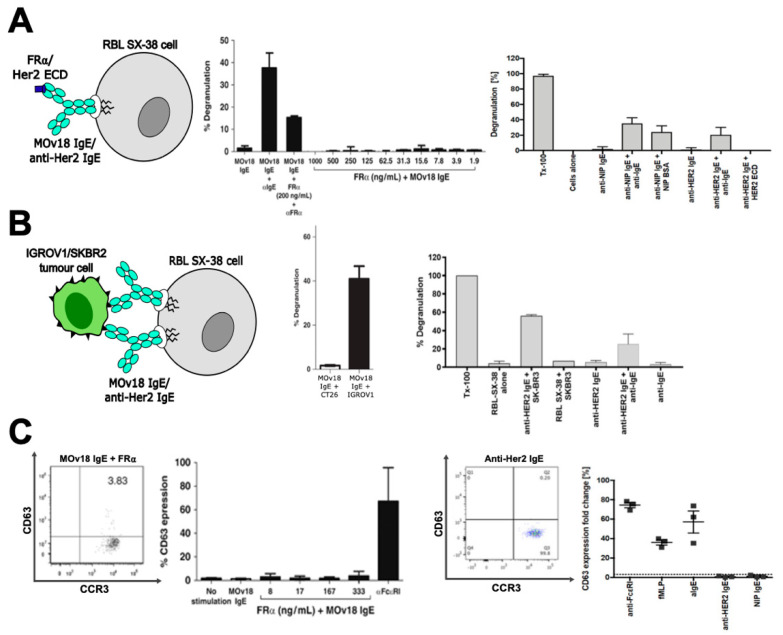
In vitro safety assessments of anti-cancer IgE antibodies. (**A**) RBL SX-38 mast cell degranulation was triggered by MOv18 and anti-Her2 (trastuzumab) IgE antibodies when cross-linked by polyclonal anti-IgE antibody or multivalent antigen or antigen complexes, but not by the antibody alone or when incubated with monovalent recombinant antigen. (**B**) Degranulation was also triggered by incubation of IgE-sensitised RBL SX-38 cells with high numbers of tumour-associated antigen-expressing cancer cells. (**C**) Basophil activation (measured as upregulation of CD63 expression by basophil activation test, BAT) was not triggered following incubation of whole blood samples with MOv18 and anti-Her2 (trastuzumab) IgE antibodies. Activation by positive control immune stimuli (anti-FcεRI, anti-IgE and fMLP) was demonstrated. Adapted from Rudman et al. [49] and Ilieva et al. [36].

A range of approaches has been employed to carry out in vitro assessment of potential hypersensitivity to therapeutic IgE antibodies (Figure 2). First, degranulation assays, using human FcεRI-expressing RBL SX-38 mast cells demonstrated that tumour-associated antigen-specific IgE antibodies, MOv18 and anti-Her2 (trastuzumab) IgE, did not induce RBL SX-38 degranulation unless cross-linking was achieved by polyclonal anti-IgE, or in the case of MOv18 IgE, by the formation of an immune complex with recombinant FRα antigen and anti-FRα polyclonal antibody. Degranulation was not triggered by either antibody in the presence of their specific monomeric antigen, even at concentrations of FRα well above those found in the sera of patients with ovarian cancer [36,47,49]. Furthermore, despite the presence of naturally shed FRα, only background levels of degranulation of MOv18 IgE-primed cells were induced by ovarian cancer patient sera [49]. To assess the possibility of cross-linking by circulating tumour cells, RBL SX-38 cells sensitised with candidate IgE were incubated with tumour-associated antigen-expressing cells. Degranulation was not triggered by MOv18 IgE when E:T cell ratios were below those recorded in patient blood. However, in the presence of high numbers of IGROV1 or SKBR3 breast tumour cells, degranulation was triggered by MOv18 and trastuzumab IgE, respectively [36,49].

Furthermore, the basophil activation test (BAT), normally applied in the field of allergy to detect hypersensitivity to a variety of allergens in patient blood, was employed to monitor basophil activation in unfractionated human blood following incubation with MOv18 IgE or trastuzumab IgE. Although basophils were activated by immune stimuli (such as anti-FcεRI, anti-IgE and the chemotactic peptide formyl-methionyl-leucyl-phenylalanine, fMLP) neither MOv18 or trastuzumab IgE induced basophil activation [36,49]. This was also the case for MOv18 IgE when co-incubated with exogenous recombinant FRα antigen or when blood samples from ovarian cancer patients with detectable circulating FRα were stimulated [49]. More recently, the BAT has been utilised for a larger study of propensity for hypersensitivity to MOv18 IgE [79] (see Section 3.5.2).

Overall these studies surveilling in vitro and ex vivo mast cell and basophil degranulation suggest that IgE, in the absence of cross-linking stimuli, does not lead to type I hypersensitivity reactions and thus supported further safety evaluations of candidate IgE therapeutics in animal models.

### 3.3. Pre-Clinical In Vivo Safety Studies of Anticancer IgE Therapeutics

**Figure 3:**
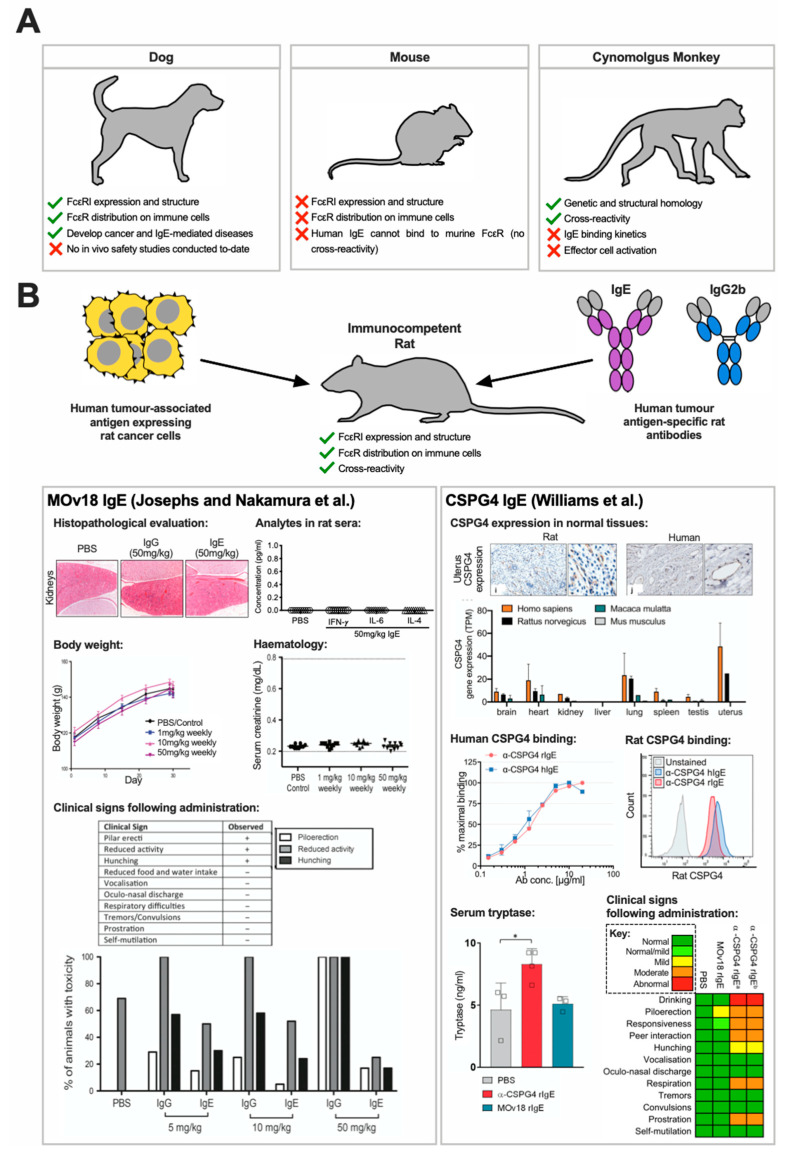
In vivo models for safety evaluations of IgE therapeutics. (**A**) IgE receptor expression and structure and distribution on immune cells, IgE binding cross-reactivity and kinetics, and IgE-associated biology across a number of species was compared with human to select the most appropriate model. (**B**) Due to similarities in IgE biology between rat and human, immunocompetent rats were used to study the safety of rat surrogate antibodies. Josephs et al. demonstrated safety of rat MOv18 IgE in immunocompetent rats challenged with human FRα-expressing CC531 cells. With treatment there were no significant changes in off-target organs (assessed by histopathology; representative images from kidneys shown), cytokines associated with hypersensitivity or cytokine storm, animal body weight and blood chemistry or haematological observations (serum creatine measurements shown). Furthermore, despite observations of some mild clinical signs of toxicity, these were short-lived and equivalent to those observed the counterpart rat MOv18 IgG antibody. Similarly, Williams et al. confirmed comparable expression profiles of human and rat CSPG4 on normal tissues (assessed by immunohistochemistry (representative images from uterus shown) and gene expression) and cross-reactivity of rat anti-human CSPG4 IgE to both human CSPG4 (on human melanoma A2058 cells) and rat CSPG4 (on rat glioma C6 cells). Following treatment with rat anti-CSPG4 IgE, mild elevation in serum tryptase was observed alongside observations of transient and mild clinical signs of toxicity (irrespective of rats having been challenged with human CSPG4-expressing CC531 cells (a) or untransfected CC531 cells (b)). Adapted from Josephs and Nakamura [67] and Williams et al. [83]. * *p* < 0.05.

Selecting appropriate in vivo models to assess the safety of IgE antibody immunotherapy for cancer is crucial (Figure 3). Most often, murine models are used to conduct pre-clinical safety evaluations of therapeutic antibody candidates. However, the mouse immune system does not fully represent the IgE biology in humans. First, the murine FcεRI is limited to expression of the tetrameric form (αβγ2), while in humans, both the trimeric (αγ2) and tetrameric forms are expressed. Second, and more critically, human IgE cannot bind to murine FcεRs [80,81] and murine FcεRI is only expressed on mast cells and basophils, whereas in humans, this expands to other crucial effector cells such as eosinophils, DCs, monocytes and macrophages [81,82]. Murine models are therefore not particularly suitable for assessment of human IgEs. Employing in vivo models that are immunologically relevant and more congruent to the human IgE biology is essential, especially when assessing the safety and toxicity implications of IgE-based therapeutics. For this purpose, a variety of animal models have been considered to-date, such as primates (i.e., cynomolgus monkeys), dogs and rats.

#### 3.3.1. Cynomolgus Monkey

Primates, such as cynomolgus monkeys, are also often considered for toxicity studies. Daniels et al. was the first to conduct a pilot experiment to consider the safety profile of a fully-human anti-Her2 (C6MH3-B1) IgE antibody in cynomolgus monkeys [47]. Following a single intravenous infusion of either 2.4 µg/kg or 80 µg/kg C6MH3-B1 IgE, over the course of one week, no changes in eating habits or general health were observed and no systemic reactions or other adverse events occurred (Table 3). However, the binding kinetics of human IgE on non-human primate FcεR-expressing immune cells was unknown.

Saul et al. reported that although human IgE cross-reacts with cynomolgus monkey immune effector cells, the binding kinetics of human IgE to primate and human effector cells differed. Binding of human IgE to primate and human peripheral blood lymphocytes (PBLs) was not comparable, unless at the highest concentration, and human IgE dissociated from monkey PBLs faster than from human PBLs. These differences may result in human IgE antibodies engaging less effectively with effector cells in cynomolgus monkeys in vivo, meaning that it may not be possible to accurately assess the effects of human IgE therapeutics in these animals. Indeed, the capacity of cynomolgus monkey PBMCs to mediate antibody-dependent cell-mediated cytotoxicity and phagocytosis (ADCC/P) against FRα-expressing IGROV1 cells, was evaluated ex vivo using the human anti-FRα MOv18 IgE. At a low concentration of MOv18 IgE, cynomolgus monkey PBMCs were significantly less efficient in killing compared to human PBMCs, although the efficiency did reach comparable levels when a higher concentration of MOv18 IgE was used. Additionally, cytokines detected in the supernatants of PBMC-mediated killing differed between human and primate, pointing to further potential differences in how effector cells of human and cynomolgus monkey origin may be activated by antigen-specific human IgE. Thus, despite the genetic similarities and predicted structural homology of FcεRI between primates and humans, due to the differences in IgE binding kinetics, cynomolgous monkeys may not accurately predict the clinical safety and toxicity studies of IgE therapeutic candidates [84].

#### 3.3.2. Canine

Dogs may be an alternative species for the in vivo study of anticancer IgE antibodies, as, like humans, they can develop cancer, such as spontaneous mammary carcinoma [85], and suffer from IgE-mediated disease, such as atopic dermatitis or food allergies [86]. Furthermore, dogs have a similar FcεR expression and distribution as humans and similar immune cell activation [86,87]. Therefore, candidate IgE therapeutics could undergo testing in dogs, in order to accelerate drug development for humans, while benefiting veterinary cancer patients as well. This “comparative oncology” approach for anticancer IgE antibodies has been initiated by the development of caninised anti-EGFR IgE (cetuximab variable regions with canine IgE Fc domains) which has undergone in vitro functional assessment [37], with the aim of ex vivo and in vivo efficacy and safety studies in the future.

#### 3.3.3. Rat

As described in Section 2.2, rats are considered a more immunologically-relevant animal model of human IgE biology, due to similar expression and distribution of IgE receptors [55]. Pre-clinical safety studies for monoclonal antitumour IgE antibodies intended as therapeutic candidates have been performed in immunocompetent rat models [32,67,83].

Josephs and Nakamura conducted safety and toxicity evaluations of MOv18 IgE, in an immunocompetent surrogate rat lung tumour metastases model (Table 3). Upon rat MOv18 IgE (MOv18 rIgE) treatment (weekly doses of 5, 10 and 50 mg/kg), animals displayed only mild indications of toxicity, such as subdued but responsive behaviour, pilo-erection and hunching. However, these responses only lasted for 10–15 min and were also observed in rat MOv18 IgG-treated animals. None of the rats in any treatment group experienced any symptoms that would constitute an anaphylactic response. When tissues from these rats were evaluated for potential off-target toxicities, no pathological lesions were observed in the spleen, lymph nodes, liver, kidney, colon or brain (even with doses of up to 50 mg/kg MOv18 rIgE). Moreover, there was no effect on body weight, serum blood chemistry or haematological parameters including creatinine, and white blood cell counts in comparison with PBS treatment. Despite detecting rat anti-human FRα antibodies across all tumour-bearing animals, and rat anti-drug antibodies (ADA) in the serum of one out of ten rats treated with 10 mg/kg and one out of ten rats treated with 50 mg/kg, overall, the study demonstrated that there were no clinical, histopathological or metabolic toxicities [67]. Furthermore, although there was upregulation of TNFα in the serum of IgE-treated rats, in comparison to PBS-treated rats, other cytokines indicative of allergic inflammation (IL-4) and cytokine storm (IL-6 and IFN-γ), were not detected. In conclusion, using this model, the efficacy and safety of MOv18 IgE were demonstrated simultaneously: IgE-treated animals did not suffer from toxicities, cytokine storm or allergic reactions, suggesting that administration of MOv18 IgE can be safe and supporting translation of the therapeutic candidate to clinical testing in man.

Similarly, in a more recent study, Williams et al. employed a WAG rat model to study the safety of an anti-chondroitin-sulphate proteoglycan 4 (CSPG4) IgE antibody, which is a promising candidate for treating melanoma, glioblastoma and subsets of breast carcinomas [88] (Table 3). First, through immunohistological analysis, Williams et al. showed that human CSPG4 and the rat orthologue had comparable expression profiles in normal tissues, making the model an appropriate tool for proof-of-concept safety evaluations of anti-CSPG4 treatments [83]. Furthermore, surrogate rat anti-CSPG4 IgE (CSPG4 rIgE) was engineered, with variable regions derived from the murine clone 225.28, which recognises the rat orthologue of CSPG4, making the rat model suitable to assess antigen cross-reactivity, while simultaneously studying immune cell engagement by the rat Fc domains of CSPG4 rIgE [35,38,83]. Animals that received a single dose of 10 mg/kg CSPG4 rIgE exhibited only transient mild to moderate adverse signs (increased drinking, laboured respiration, prostration, piloerection, and reduced responsiveness and peer interactions) within 5 min of injection which resolved within 30 min. Clinical signs were observed regardless of prior inoculation with syngeneic rat cancer cells (CC531) with or without human CSPG4 surface expression. These symptoms were accompanied by mild elevation in serum tryptase concentrations, but no change in angiotensin II or cytokines associated with allergic reactions and cytokine storm. Additionally, repeated administration of 5 mg/kg anti-CSPG4 rIgE on days 1, 2, 4, 7 and 14, was deemed non-toxic as demonstrated by no changes in animal body weight, liver or kidney functions, or blood cell counts [83]. Overall, administration of CSPG4 IgE antibody appeared to be well tolerated. Taken together with the similar study of MOv18 IgE, the administration of IgE antibodies targeted against tumour-associated antigens did not lead to any severe adverse events, particularly anaphylactic reactions, in immunologically relevant rat models.

In conclusion, to establish the safety profiles of IgE-based treatments, multiple in vitro and in vivo assessments have been conducted, with studies to-date indicating that IgE treatment does not cause hypersensitivity type I reactions or other significant adverse reactions. These findings provided further evidence for the potential safe administration of IgE-based therapeutics in humans and greater confidence in the translation of these candidates to the clinic.

### 3.4. IgE Antibodies Are Heavily Glycosylated

**Figure 4:**
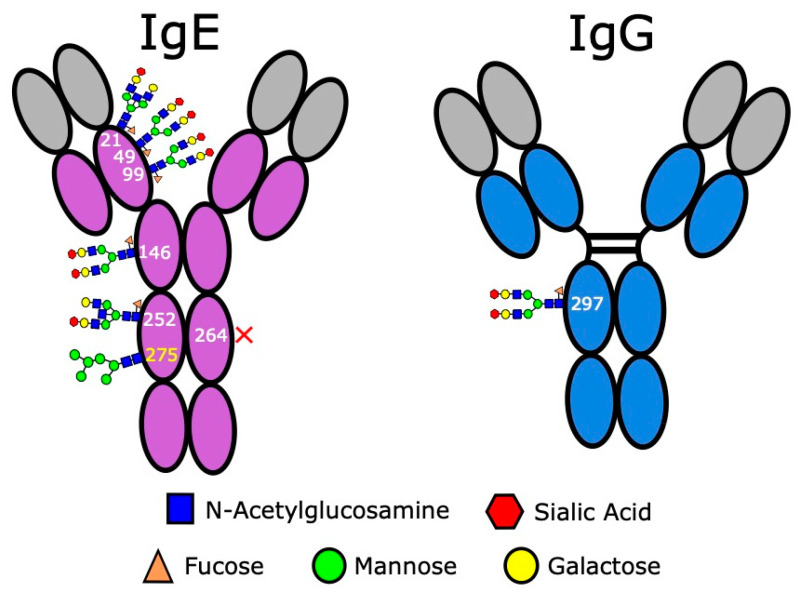
IgE is a heavily glycosylated molecule, with 7 documented N-glycan sites. Site N275 (highlighted yellow) is occupied by a simple-type oligomannose glycan. Site 264 has been found to be consistently unoccupied (indicated by red cross). All remaining sites are occupied by complex type glycans. Glycosylation patterns shown here are adapted from Plomp et al. [89]. In comparison, IgG has one detectable glycan in its Fc region, and in around 20% of IgG molecules there may additionally be glycosylation in the Fab region. In both antibody classes there is evidence that pathophysiological states and individual variation can result in differential glycosylation, so glycan composition shown here is representative rather than indicative of specific glycosylation patterns.

As the most heavily glycosylated antibody class in humans (Figure 4), with a total of six occupied N-glycan sites [89], the glycosylation of IgE must be carefully considered when designing and producing therapeutic antibodies of this class. In human immunoglobulins, glycans influence a number of properties, including correct expression under native conditions, water solubility and aggregation, and structural conformation [90]. While IgE glycans are required for native secretion, there is much debate surrounding their exact involvement in IgE structure and function, and, generally, most existing studies suggest that out of the six IgE glycans, only the simple oligomannose N275 glycan—considered analogous to the sole IgG Fc glycan at N297—is essential for IgE effector functions [91]. Loss of the N275 glycan is shown to abrogate FcεRI binding and consequently abolish downstream effector functions [92], but conversely most evidence suggests the remaining complex-type glycans can all be safely removed without significant impact on IgE structure or function [91,93]. However, a recent study demonstrated that removal of sialyic acid from IgE attenuated effector functions and reduced anaphylaxis in models of allergy [94]. Therefore, safety issues surrounding IgE glycosylation should include further studies in the context of anticancer mechanisms, as well as considerations for choice of cell line expression system and the addition of foreign glycans, for minimising immunogenicity.

Most current expression systems for production of pharmaceutical biologics are of non-human origin. The majority of biologics are produced in Chinese Hamster Ovary (CHO) cell lines, with the remainder from either NS0 or Sp2/0 lines [95]. However, a potential issue with non-human expression systems is the addition of non-human glycans and linkages which can promote severe reactions in patients described for IgG antibody therapies [96]. In some instances, the presence of foreign glycans on monoclonal antibodies (mAbs) can result in severe and potentially fatal immune responses, including cytokine release syndrome, serum sickness and anaphylaxis [97]. Perhaps the most well-known example is that of the galactose-α1,3-galactose linkage (α-Gal), a mammalian glycan not found in higher apes and humans. α-Gal hypersensitivity (α-Gal syndrome) is a condition typically associated with an allergic response, including anaphylaxis, to mammalian food products in individuals previously sensitised by tick bites [98,99]. A similar hypersensitivity response was noted in patients undergoing first-time administration of the EGFR-specific mAb, cetuximab, later determined to be predominantly occurring in patients with preexisting IgE sensitised towards α-Gal, although a small number had no detectable specific IgE [99,100]. Glycan analysis of cetuximab, which is commonly produced in Sp2/0 cells [96,99], found approximately 21 distinct glycan structures, of which 30% carried one or more α-Gal residues [101]. Future studies of therapeutic IgE antibodies may include similar glycan analysis.

Another expression system used in IgE expression is plants [39,102], such as *Nicotiana benthamiana*, which can introduce the similarly immunogenic plant glycans α1,3-fucose and β1,2-xylose [96]. Some studies have suggested as many as 50% of healthy individuals may already possess antibodies against these glycans [103]. However, the degree of immunogenicity of these glycans is subject to debate. A study investigating the immunogenicity of plant-expressed therapeutics found no hypersensitivity response towards glycans on plant-derived particle-based influenza vaccines across 280 trial subjects [104]. Supporting this, Shaaltiel and Tekoah claimed that there is little evidence for hypersensitivity to plant-derived biologics; however, their plant-derived therapeutic for Gaucher’s disease, taliglucerase alfa, has a comparable incidence of hypersensitivity to cetuximab at 29% [105], suggesting instead that plant glycans have a similar degree of immunogenicity to mammalian glycans. Additionally, while plant-derived therapeutic antibodies, such as the experimental Ebola drug ZMapp [106], are under development, little investigation seems to have been directed towards potential hypersensitivity responses. Therefore, we would suggest that plant-expressed anticancer IgE therapeutics are not entirely risk-free and some degree of caution should still be taken.

Prior to treatment with therapeutic mAbs derived from non-human expression systems, it may be advisable to monitor patients for hypersensitivity. It must be noted, however, that presence of specific antibodies will not necessarily correlate to clinical allergy, for example, a proportion of patients undergoing hypersensitivity towards α-Gal on therapeutic antibodies had no prior evidence of sensitised IgE [98,100,107]. Blood tests, such glycan-specific IgE assays, or basophil activation tests (discussed in more detail in Section 3.5.2), may be useful tools for predicting risk [107] but lose value if patients are not previously sensitised. Alternatively, a safer option may be to simply select human-compatible cell lines, such as CHO cells. CHO-based expression systems are already a staple in the pharmaceutical industry [96] and, importantly, do not express alpha-1,3-galactosyltransferase, thereby eliminating expression of α-Gal. Supporting this, cetuximab produced in CHO cells did not elicit hypersensitivity responses, even in patients sensitised to Sp2/0-derived cetuximab, owing to lack of α-Gal [100]. Human-derived cell lines, such as human embryonic kidney cells (HEK293), are also seeing use in production of therapeutic IgE candidates [36]. However, a potential risk with these cells is the high degree of heterogeneity amongst glycoproteins, with as many as 30 different glycoforms detected in HEK293-derived IgE [102]. While there may not currently be any indications for roles of individual glycans or sugar moieties in driving antitumour IgE effector functions, glycan composition may still need to be considered, for instance, high mannose content is associated with rapid clearance from serum, potentially requiring higher dosage for efficacy which in turn may increase risk of adverse events [108]. Ultimately, the choice of expression system should be carefully considered to optimise glycan profiles in relation to the safety of IgE therapies in humans.

### 3.5. Clinical Tools to Predict and Monitor Safety

Tools that are used more widely in the allergy field, such as skin testing and basophil activation test (BAT), may be useful in predicting and monitoring safety of anticancer IgE therapeutic candidates.

#### 3.5.1. Skin Tests

The general principle of the skin tests, such as the skin prick test (SPT) and intradermal skin test (IDST), is the use of cutaneous skin reactivity as a surrogate for sensitisation within a target organ. The introduction of an allergen to the forearm skin using a lancet, as is the case for SPT, or a thin hyperdermic needle within the superficial dermis for IDST, can thereafter cause the cross-linking of specific IgE bound to mast cells, resulting in mast cell degranulation and the release of histamine and other mediators to the cutaneous skin layer. Within 15 to 20 min the wheal and flares produced on the skin can be quantified and a result determined [109]. A positive SPT result is determined by a wheal of greater than 3 mm diameter, and once collated with a patient’s clinical history, can be used to confirm sensitivity to an allergen. A positive IDST result is determined by a wheal of at least 7 mm, and 2 mm greater width that that produced by the control [110]. The positive predictive value (PPV) of SPT can vary between allergens: PPV of allergic rhinitis has been shown to be 97–99% [111], whereas for food allergens, such as cow’s milk and hen’s eggs, reported PPVs are 76% and 89%, respectively [112]. Skin testing can also play an important role in identifying patients at risk of allergic reactions to chemotherapy drugs. A study of the combination of SPT and IDST looking at hypersensitivity to platinum salts found that 66% of at risk patients were identified through positive skin tests. This combination study and others assessing SPT and IDST have shown that the negative predictive value (NPV) for these skin tests is 92%, 99% and 98.5%, respectively [113,114,115]. Likewise, in a relatively small study investigating platin chemotherapies, carboplatin and oxaliplatin, with a combination of SPT and IDST, PPV was found to be 100% and 75%, respectively [116].

Limitations for skin testing can include difficulties in identification of wheal size, such as in patients with extensive eczema, dermographism, urticaria or following the use of anti-histamines. Patients with elevated serum IgE resulted in increased numbers of false positive results, which could mean that borderline positive results may not be clinically relevant [103]. To counteract these concerns, skin tests incorporate a positive histamine control and a negative control using an allergen-free lancet to exclude dermographic urticaria [109].

Skin testing offers a standardised approach for assessing the IgE-mediated sensitisation to an allergen. The test can provide an objective confirmation of allergen sensitivity through a rapid, reproducible, and minimally invasive procedure [109]. Standardised European recommendations for the SPT have existed for many years [117] and subsequently the Global Allergy and Asthma European Network (GA^2^LEN) have sought to standardize the procedure further, allowing for better comparison and comparability between various countries [109]. Nevertheless, skin tests do pose a minute, but not impossible, risk of triggering anaphylaxis, and may not accurately predict allergic responses triggered at other sites, such as in the circulation.

#### 3.5.2. Basophil Activation Test (BAT)

**Figure 5:**
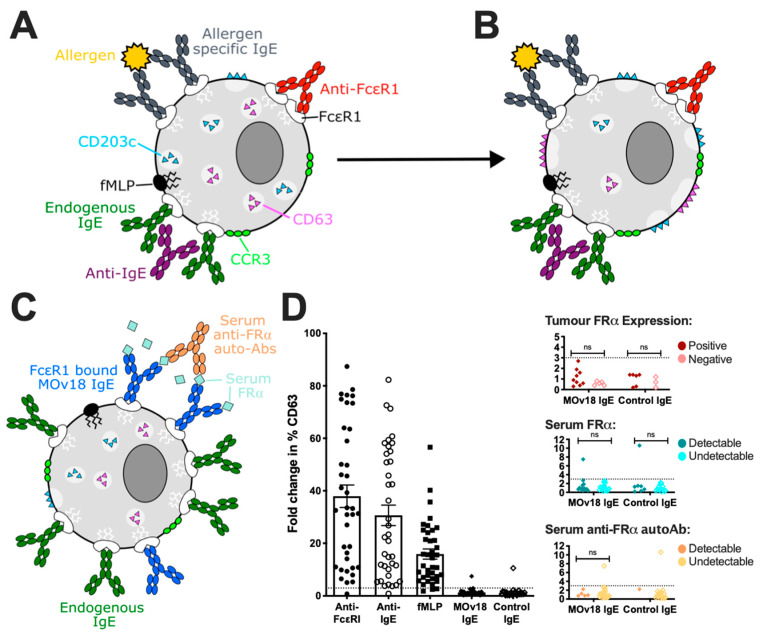
Basophil activation test (BAT). (**A**) Basophils can be identified in unfractionated whole blood samples by cell surface markers, such as CCR3 and CD203c. (**B**) Surface expression of CD63 and CD203c is upregulated in activated basophils through the fusion of intracellular vesicles, via both IgE- (anti-FcεRI, anti-IgE and allergen cross-linking of specifc IgE) and non-IgE-mediated (fMLP) stimuli. Adapted from Bax et al. [11]. (**C**) Circulating FRα anti-FRα autoantibodies found in ovarian cancer patient sera may form immune complexes with MOv18 IgE triggering basophil activation. (**D**) Ex vivo basophil activation (>3.0 fold change in % CD63 expression) was measured following incubation with anti-FcεR1, anti-IgE, fMLP stimulation, but not triggered by MOv18 and control IgE antibodies, in all but one patient. Activation by MOv18 IgE, or lack thereof, was irrespective of patient tumour FRα expression, or the presence of FRα and anti-FRα autoantibodies in patient sera. Adapted from Bax et al. [79].

Basophils are a key cell involved in IgE-mediated type I hypersensitivity reactions. The basophil activation test (BAT) has been widely utilised in the allergy field as a methodology to study hypersensitivities to a number of stimuli, and more frequently alongside existing standard of care testing. Patient groups investigated include those with reactivity to foods [118,119,120,121], drugs [122,123,124,125] and venoms [126,127,128]. The BAT is a flow cytometric method performed on basophils in unfractionated whole blood that are typically identified by antibodies for markers such as CCR3, CD123, CD203c and IgE. Ex vivo basophil activation is measured as upregulation of activation markers on the cell surface, such as CD63 or CD203c. Commonly used positive controls trigger basophil activation through IgE-dependent mechanisms (such as incubation with anti-FcεRI and anti-IgE antibodies) and IgE-independent pathways (i.e., fMLP) [129] (Figure 5A,B).

A significant advantage of the BAT is that through a relatively noninvasive blood assay carried out ex vivo, basophil activation can be measured and consequentially the propensity for type I hypersensitivity to an allergen can be predicted. Unlike conventional oral food challenging (OFC) methodologies, BAT does not increase the risk of anaphylaxis, as the patients are not directly challenged with the allergen. Beneficially, this allows the BAT to be used in combination with many existing methodologies, or to reduce the total number of tests overall, or as a confirmatory tool. Whole blood is used in this assay, therefore reducing the processing and handling of the cells required for isolation, which could otherwise impact the activation of the basophils. The use of the whole blood also acts to preserve all molecules found within the blood, more accurately capitulating the activation that would be observed in vivo. Specifically, and in the case of allergies, this includes the presence of presensitised IgE antibodies found on basophil cell surface receptors, and also any molecules involved in the formation of allergen/drug-immune complexes triggering basophil activation.

The application of the BAT outside of the allergy field is a relatively new and exciting development. Recent papers have sought to apply the BAT to the oncology field [130,131,132,133,134]. These studies have explored the application of the BAT in individuals with broad clinical histories, namely, to predict hypersensitivity and monitor desensitisation. The BAT acts as a biomarker of drug hypersensitivity reactions (DHRs), and provides a method to monitor the safety and efficacy of rapid drug desensitisation (RDD) whereby exposure to the hypersensitivity-inducing drug, in gradually increasing doses and for longer time periods, is employed in an effort to achieve tolerance to the therapy [130].

Given the utility of BAT in other fields, and the importance of circulating basophils when considering the perceived risk of anaphylaxis following administration of anticancer IgE antibodies, the technique has been most recently been employed in the development of these therapeutic candidates. Having identified CCR3-positive basophils in whole blood samples from cancer patients, capacity for activation was confirmed by stimulation with anti-FcεR1, anti-IgE and fMLP [36,79]. Furthermore, the presence of IgE-receptors, through which basophil activation could be triggered, was demonstrated by CD63 upregulation following incubation with exogenous IgE and multivalent antigen [79]. This study then showed that in 41 out of 42 of cancer patients studied, ex vivo stimulation with the anticancer IgE (MOv18) did not trigger activation of basophils. This observation was (i) irrespective of the prior treatment history of the patient; (ii) despite a patient having already raised serum tryptase and total IgE concentrations, which could suggest underlying allergic disease or sensitivity; and (iii) irrespective of the patients’ tumour FRα expression, or presence of FRα or anti-FRα autoantibodies in the circulation (molecules which may hypothetically form an immune complex with MOv18 IgE, triggering basophil activation) (Figure 5C). In the one patient where basophils were activated by MOv18 IgE, circulating FRα was present, but no anti-FRα autoantibodies. Furthermore, basophils from this patient were also activated by the non-FRα-specific control IgE, suggesting that basophil activation in this individual may have been triggered by another mechanism such as a humoral response directed toward IgE. Similarly, basophils were not activated by an anti-Her2 (trastuzumab) IgE [36], although a larger number of patient blood samples are yet to be stimulated with this therapeutic candidate to indicate whether the rate of reactivity is comparable to that observed for MOv18 IgE.

Basophil identification and the propensity of these cells for immune activation was also confirmed in a cohort of 53 ovarian cancer patients with a wide range of tumour histologies and treatment histories [11], suggesting that the use of BAT in evaluating hypersensitivity to an IgE therapy is applicable to a broad spectrum of patients. Individuals for whom it may not be possible to obtain an informative BAT result include those who had received prolonged oral corticosteroid treatment, which was associated with a significant ablation of the basophil population. Another small subset of “non-responders” have basophils that are not reactive to positive control stimuli, meaning activation, or lack thereof, following stimulation with test antibody cannot be accurately interpreted. However, the prevalence of these samples in the cancer patient cohort is low (5.8%) and comparable to that reported for individuals with allergic disease [11,118,120].

The BAT is yet to be standardised and approved for use as a standalone diagnostic tool, but it has proved useful to study hypersensitivity reactions across many patient groups. In particular this assay has been studied, alongside other clinical parameters, as a safety monitoring companion for the development of therapeutic IgE candidates, as in a first-in-class phase I trial of MOv18 IgE.

#### 3.5.3. First-in-Human Study of MOv18 IgE

The first-in-class clinical study of an anticancer IgE therapeutic candidate is currently underway (MOv18 IgE; NCT02546921). This phase I trial has incorporated significant safety precautions due to the perceived risk of anaphylaxis with IgE therapy. Skin prick testing prior to each infusion as well as BAT at baseline and intervals in the treatment regimen were implemented in an effort to predict and monitor type I hypersensitivity to MOv18 IgE. To date, MOv18 IgE treatment has been well tolerated in most individuals [135]. The most common toxicity was urticaria, although this was manageable and not associated with systemic symptoms. In the one patient who experienced anaphylaxis, ex vivo basophil activation by MOv18 IgE was observed in the BAT performed with blood from this patient prior to any exposure. Therefore, the baseline BAT was subsequently used in this MOv18 IgE trial to exclude recruitment of further patients with reactive basophils would receive MOv18 IgE treatment [135]. Initial results support the safety profile of anticancer IgE therapy, and preliminary evidence for antitumour activity has been reported.

## 4. Future Development of IgE Immunotherapy

Despite the extensive and successful development of pipelines to engineer and produce IgE antibodies for pre-clinical study, including GMP process development of MOv18 IgE for its phase I clinical trial, further considerations for production for clinical translation must be explored. Important strides have been made in producing IgE antibodies in mammalian expression systems and improving IgE antibody yields [32,35,36,38]. Future work involves further expansion of protein production techniques for larger scale antibody generation and good manufacturing practice (GMP) considerations of the quality of the preparations produced. In particular, these may include the study of glycosylation of IgE antibodies (as described above in Section 3.4), as well as the impact of any contaminants that may be present following manufacturing processes of IgE [136,137].

Further development of IgE antibodies as therapies for cancer will include consideration of a variety of tumour-associated antigens, such as those already targeted with some clinical success by IgG antibodies, as well as novel targets as they are discovered and validated. The application of pre-clinical efficacy and safety studies to phase I clinical trials of IgE antibodies, as was used in the development of MOv18 IgE, have paved the way and will continue to contribute to an expansion of the understanding of the safety profile of these agents.

## Figures and Tables

**Table 1:** In vitro studies of IgE antibody therapy efficacy.

Target of IgE	IgE Species	Direct Effect	ADCC	ADCP	Degranulation ^1^	Antigen Presentation	Refs.
Human FRα	Mouse/human chimeric	-	✓	✓	✓	-	[29,30,42,49]
Human FRα	Mouse/rat chimeric	-	✓	✓	-	-	[32]
Human Her2	Humanised	✓	✓	✕	✓	-	[36,43]
Human EGFR	Mouse/human chimeric	✓	✓	✕	✓	-	[44]
Human Her2	Human	-	-	-	✓	✓	[47]
Human PSA	Mouse/human chimeric	-	-	-	✓	✓	[48]
Human CD20	Mouse/human chimeric	-	✓	✕	-	-	[45]
Human colorectal cancer	Human	-	✓	✕	-	-	[7]
Human EGFR	Canine	-	✓	✕	-	-	[37]
Human Her2	Human ^2^	-	-	-	✓	-	[39]
Human SF-25	Mouse/human chimeric	-	✓	✕	-	-	[46]

^1^ When cross-linked by polyclonal anti-IgE antibody, multimeric antigen or antigen-expressing tumour cells; ^2^ Plant-derived. ✓: Measured; ✕: Not measured; - Not tested; ADCC: antibody-dependent cell-mediated cytotoxicity; ADCP: antibody-dependent cell-mediated phagocytosis.

**Table 2:** In vivo pre-clinical assessments of IgE antibody therapy efficacy.

Target of IgE	IgE Species	Tumour Model (Tumour Site)	Animal Model ^2^	Antibody Injection Route	Findings ^1^	Ref.
Gp36 of MMTV	Mouse	H2712 mouse mammary carcinoma (s.c. and i.p.)	Syngeneic C3H/HeJ mice	i.p.	Inhibited the development of tumours	[50]
COLO 205	Mouse and mouse/human chimeric	Human colorectal COLO 205 carcinoma (s.c.)	SCID mice	i.v	Inhibited the growth of established tumours	[51]
Human FRα	Mouse/human chimeric	IGROV1 human ovarian carcinoma (s.c.)	PBMC engrafted SCID mice	i.v.	Greater anti-tumour activity than IgG1	[31]
		HUA patient derived ovarian carcinoma donor mice ascites (i.p.)	PBMC engrafted nude mice	i.p.	Prolonged mouse survival ^1^	[29]
			PBMC ^2^ engrafted nude mice	i.p.	Prolonged mouse survival ^1^	[42]
			U937 (+/− IL-4 treatment) engrafted nude mice	i.p.	Prolonged mouse survival ^1^	[30]
Human Her2	Human	Her2-expressing D2F2/E2 mouse mammary carcinoma (i.p.)	hFceR1α Tg BALB/c mice	i.p.	Prolonged mouse survival	[47]
Human MUC1	Mouse/human chimeric	MUC1-expressing 4T1 mouse mammary carcinoma (s.c.)	hFceR1α Tg BALB/c mice	s.c	Reduced tumour growth by 25–30%	[45]
Human PSA	Mouse/human chimeric	PSA-expressing CT26 mouse colon adenocarcinoma (s.c.)	hFceR1α Tg BALB/c mice	s.c	Prolonged mouse survival	[48]
Human FRα	Mouse/rat chimeric	FRα-expressing CC531 rat colon adenocarcinoma (lung mets. from i.v. injection)	Immuno-competent WAG rat	i.v.	Superior efficacy compared to mouse/rat IgG2b	[32]

^1^ The role of monocytes in efficacy was observed (see Section 2.3); ^2^ Some PBMCs were depleted of or reconstituted with monocytes.

**Table 3:** In vivo safety and toxicity evaluations of therapeutic IgE candidates.

Therapeutic Antibody	Antibody Target	Dose	Model Species	Findings	Ref.
C6MH3-B1 IgE	Human Her2	2.4 and 80 µg/kg	Cynomolgus monkey	No systemic reactions or adverse events observed.	[47]
MOv18 IgE	Human FRα	5, 10, 50 mg/kg	Rat	No clinical, histopathological or metabolic signs of a type I hypersensitivity reaction, but some mild responses such as piloerection and hunching which were observed to the same degree in IgG-treated rats.	[67]
CSPG4 IgE	Human and rat CSPG4	10 mg/kg (for immediate reactions); 5 mg/kg (for long-term safety)	Rat	Only transient mild to moderate adverse events within 5 min which resolved by 30 min, accompanied by mild elevation in serum tryptase. No long-term toxicity observed.	[83]
